# Did COVID‐19 Pandemic Change the Course of Multiple Sclerosis? A Retrospective Eight‐Year Study

**DOI:** 10.1002/hsr2.71649

**Published:** 2025-12-14

**Authors:** Przemyslaw Puz, Marta Konieczna, Katarzyna Maciejowska, Zofia Tarnawska, Zofia Nowakowska, Mateusz Glodny, Anetta Lasek‐Bal

**Affiliations:** ^1^ Department of Neurology, Faculty of Health Sciences in Katowice Medical University of Silesia Katowice Poland; ^2^ Upper‐Silesian Medical Centre Silesian Medical University in Katowice Katowice Poland; ^3^ Students' Scientific Association, Department of Neurology, Faculty of Health Sciences in Katowice Medical University of Silesia Katowice Poland

**Keywords:** COVID‐19 pandemic, COVID‐19 vaccination, infections, multiple sclerosis, predicting factors, therapy change

## Abstract

**Background and Aims:**

The COVID‐19 pandemic was a special situation for patients treated with disease‐modifying therapies (DMTs). Infections may provoke disease activity and determine the choice of DMTs. Moreover, DMTs create a potentially increased risk of infections. The study aimed to assess the impact of the COVID‐19 pandemic and the importance of COVID‐19 vaccination on the course of multiple sclerosis (MS).

**Methods:**

The 8‐year single‐center retrospective cohort study compared treatment changes and infection incidence in 115 MS patients before and during the COVID‐19 pandemic, as well as COVID‐19 incidence and severity before and after COVID‐19 vaccination. A multivariate approach assessed the effects of specific factors (age, sex, disease duration, prognostic profile, disease activity before the pandemic, DMTs, and infections) on the course of MS.

**Results:**

Relapse activity was the most common reason for therapy change, with no significant differences (56.52% vs. 61.52%, *p* = 0.998) before and during the pandemic. There were no differences in the rate of infections before and during the pandemic (67.82% vs. 55.65%, *p* = 0.16). The severity of COVID‐19 courses showed a significant improvement after vaccination (*p* = 0.047). In the multivariable approach, the only significant factor associated with a higher risk of relapse was disease activity before the pandemic period (OR = 6.80, *p* = 0.010).

**Conclusions:**

The COVID‐19 pandemic did not affect the course of MS in long‐term follow‐up. The only factor associated with relapse activity during the pandemic period was disease activity in the pre‐pandemic period. COVID‐19 vaccination may be an essential tool in the care of MS patients.

## 1 Background

Predicting disease activity and disease progression in patients with relapsing remitting multiple sclerosis (RRMS) is one of the challenges in daily clinical practice and the research field. While unfavorable prognostic factors for patients' risk of disease progression and disability are known, based on which we select disease‐modifying therapies (DMTs), their predictive value is limited [1, 2, 3, 4, 5, 6]. One of the factors that provokes disease activity and determines the choice of DMTs is infections of various kinds.

The therapies that modify the course of multiple sclerosis, including the high‐efficacy therapies (HET), act on the immune system by modulating or suppressing it. The effects of DMTs on the immune system create a potentially increased risk of infections. Infections in MS patients are significant, causing a pro‐inflammatory shift in the balance of the immune system, which can cause disease exacerbation and lead to increased relapse activity and progression of disability [7, 8, 9]. In patients with MS, infections are more common than in the general population, and patients are more likely to experience severe infections [7, 10, 11].

The coronavirus disease 2019 (COVID‐19) pandemic was a special situation for patients chronically treated with DMTs. Concern arose, supported by the results of animal studies, that severe acute respiratory syndrome coronavirus 2 (SARS‐CoV‐2), due to its neurotropic nature, could exacerbate the immunopathogenesis of MS and lead to clinical MS activity. However, the results of available studies are inconclusive [12, 13, 14, 15, 16].

Most of the scientific data published to date support the safety of DMTs during the pandemic; however, data on the association of a history of COVID‐19 infection with the risk of disease exacerbations and the severity of COVID‐19 in patients are inconclusive [13, 14, 17, 18, 19]. Risk factors for a worse course of COVID‐19 identified to date include older age, male sex, presence of comorbidities, lymphopenia, recent steroid use, advanced disability, progressive forms of the disease, and anti‐CD20 therapy [19, 20].

There remains a lack of data regarding the long‐term prognosis after COVID‐19 and vaccinating MS patients against COVID‐19 [17, 19]. Previously published data on vaccine efficacy indicate that the immune response varies depending on the therapy used. Vaccination may increase the risk of disease activity, while DMTs may cause ineffectiveness of the immune response following vaccination [19].

Understanding the impact of COVID‐19 vaccination on the incidence and severity of COVID‐19 in patients with MS is crucial for optimizing patient care.

The study aimed to assess the impact of the COVID‐19 pandemic on the course of MS by comparing the frequency and causes of DMT changes before and during the pandemic. An additional objective was to evaluate the course of infection in patients with MS treated with DMTs before and during the pandemic, and to assess the importance of COVID‐19 vaccination on the course of MS.

## 2 Methods

**Figure 1:**
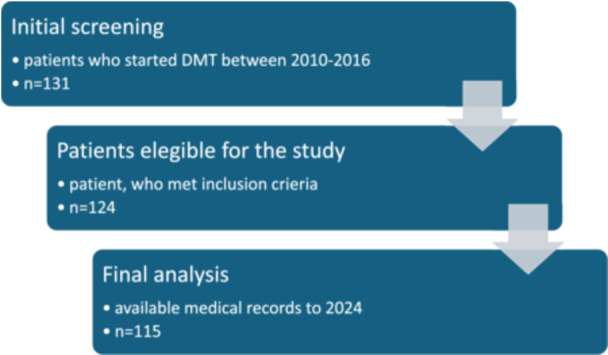
Patients' flow diagram.

This retrospective single‐center 8‐years longitudinal cohort study involved the follow‐up of 115 patients. Initially, 131 patients who commenced DMT therapy between 2010 and 2016 were screened for the study. After taking into account the inclusion criteria, 124 patients who continued therapy until 2024 were analyzed. Finally, after checking the available data, 115 patients were included in the study. Figure 1 shows the patients' flow diagram.

### 2.1 Participants

The study included a series of consecutive patients who started DMT between 2010 and 2016 and continued treatment until December 2024. The course of the disease was analyzed in the pre‐pandemic period (February 1, 2017–February 29, 2020) and during the pandemic (postpandemic) period (March 1, 2020–December 31, 2024).

The inclusion criteria were a diagnosis of RRMS according to the current McDonald criteria, the DMT duration of at least 3 years, treatment with the first DMT at the start of follow‐up, and continuation of DMT until December 31, 2024. All patients started therapy within 1 year of the diagnosis. Standard follow‐up visits occurred every 3 months.

### 2.2 Study Procedures

All patients were assessed for: demographics (age, gender), year of initial DMT, and type of second therapy in case of a change of therapy. Natalizumab, oral cladribine, anti CD20 agents, and sphingosine‐1‐phosphate receptor (S1P) modulators were considered as high effective treatment (HET). Meanwhile, interferons (INFs), dimethyl fumarate (DMF), glatiramer acetate (GA), and teriflunomide were considered as moderate treatment (platform therapies).

– The presence of unfavorable prognostic factors at the time of initiation of first DMT:

- Clinical factors: a high annual relapse rate ( ≥ 2), a short time between the first and second relapses (less than 6 months), incomplete resolution of symptoms after a relapse, and multifocal symptom presentation at the onset of disease;
- Radiological factors: ≥ 2 gadolinium enhancing (Gd+ ) lesions or at least 9 hyperintense lesions in the T2 magnetic resonance imaging (MRI) sequence, and the presence of demyelinating lesions in the spinal cord or brainstem;
- Demographic and other factors, including male gender, older age of the patient at diagnosis ( > 40 years), obesity, and nicotine use.

The presence of a minimum of five unfavorable prognostic factors was defined as an unfavorable prognostic profile.

Disease activity (occurrence of clinical relapses, confirmed disability progression (CDP), MRI activity) during each year of DMT. CDP was defined as an increase of at least 1.5 in Expanded Disability Status Scale (EDSS) from a baseline score of 0, a 1‐point increase from a baseline of 1.0–5.5, or a 0.5 increase from a baseline of at least 5.5 sustained at two or more consecutive visits separated by at least 90 days. Relapse was defined as a clinical episode lasting at least 24 h, in the absence of fever, infection, or acute concurrent illness, with a preceding 30‐day period without clinical relapse. MRI activity was confirmed by the presence of new or enlarged T2 lesions and/or Gd+ lesions.

In patients without relapses, CDP and MRI activity, No Evidence of Disease Activity (NEDA) was acknowledged. For the purposes of this study, the following definition was used: “disease activity before pandemic”, if the patient had a relapse and/or CDP and/or MRI activity in the pre‐pandemic period.

− The presence of side effects of DMT.
− Presence of acute non‐COVID‐19 infections before and after the pandemic compared to other members of patients' families (more often, less often, as often). Serious infections were defined as those requiring hospitalization.
− COVID‐19 incidence data and its course (mild, moderate, severe), and the course comparison to other family members of patients (same, milder, worse), before and after vaccination against COVID‐19.
− Vaccination against COVID‐19 data: number of doses and date of first vaccination.

Data were collected based on analysis of medical records and patient interviews.

### 2.3 Data Analysis

Analyses include the comparison of treatment changes, new medications before and during the COVID‐19 pandemic, infectious disease incidence before and during the COVID‐19 pandemic, and COVID‐19 incidence and severity before and after COVID‐19 vaccination. Estimation of the effects of specific factors on the course of MS (relapses, CDP, MRI activity) in the (post)pandemic period used the multivariate approach.

The study was conducted in accordance with the Declaration of Helsinki. The Ethics Committee of Medical of Silesia approved on January 30, 2024 (no. BNW/NWN/0052/KB1/141/I/22/23/24), with the need for written informed consent waived.

### 2.4 Statistical Analysis

A significance level of *α* = 0.05 was used to control for a 5% Type I error rate. Descriptive statistics summarized the data with continuous variables reported as median (with interquartile range (IQR:Q1–Q3), and categorical variables were described as frequencies (*n*) and percentages. Compliance of the distribution of these variables with normal distribution was verified using the Shapiro–Wilk test. The *t*‐test (for variables with normal distribution) or Wilcoxon rank‐sum test (for variables where the distribution differed from normal distribution) compared continuous variables between two independent groups. Pearson's *χ* ^2^ test or Fisher's exact test was used for categorical variables based on sample size. For dependent groups, the McNemar test (binary outcomes) or Bowker's test of symmetry (nominal variables with > 2 categories) was applied.

Logistic regression with a logit link function analyzed the effect of multiple variables (age, sex, disease duration, prognostic profile, disease activity before pandemic period, DMT, COVID‐19 infection, other infections) on binary outcomes (relapse, MRI activity, CDP). Multicollinearity was assessed using the variation inflation factor (VIF), with VIF < 5.0 indicating no multicollinearity. Confidence intervals and *p* values were calculated using the asymptotic *z*‐test approximation.

## 3 Results

### 3.1 Patients Characteristics

The study analyzed data from 115 adult patients diagnosed with MS, aged between 18 and 71 years (median age: 45 years, with IQR: 33–54 years), including 82 females (71.3%) and 33 males (28.7%). Mean disease duration was 4.10+/−0.86 years. The most common first‐line therapy was DMF—76 (66.09%) of patients, followed by INFs—19 (16.52%), GA—13 (11.30%), and teriflunomide—7 (6.08%) patients.

The unfavorable prognostic profile at the time of DMT initiation was found in 36 (31.30%) patients.

### 3.2 Treatment Change

Before the COVID‐19 pandemic, DMT was changed in 65 (56.52%) of the patients. The most common reason for a change was relapse activity—42 (72.31%) of patients. Adverse effects, including infections, were the cause of change in 8 (12.31%) patients (Table 1). New therapies that were used before the pandemic were mainly platform drugs, with 22 (33.84%) patients receiving HET (Table 1).

**Table 1:** Comparison of treatment changes, new medications, in MS patients before and during the COVID‐19 pandemic.

Characteristic	*N*	Time point		*p*
		Up to II 2020, *n* = 115	III 2020 and later, *n* = 115*	
Change of the treatment		65 (56.52%)	28 (24.35%)	< 0.001*
Main reasons for therapy change:				
Clinical relapses		47 (72.31%)	16 (61.54%)	0.998***
MR activity		9 (13.85%)	4 (14.28%)	
Side effects		8 (12.31%)	5 (17.85%)	
EDSS progression		1 (1.54%)	0 (0%)	
Pregnancy		0 (0%)	1 (3.57%)	
Conversion to SPMS		0 (0%)	2 (7.14%)	
New DMT				
DMF		23 (34.85%)	7 (25%)	0.326**
Fingolimod		10 (15.15%)	0 (0%)	0.029**
Natalizumab		9 (13.64%)	4 (14.28%)	0.753**
GA		8 (12.12%)	1 (3.57%)	0.269**
INF		7 (10.61%)	0 (0%)	0.098**
Teriflunomide		6 (9.09%)	2 (7.14%)	1.000**
Ocrelizumab		1 (1.52%)	6 (21.43%)	0.003**
Cladrybine		2 (3.04%)	3 (10.71%)	0.080**
Ozanimod		0 (0%)	1 (3.57%)	
Ofatumumab		0 (0%)	4 (14.29%)	0.008**

* Proportion test.

** Fisher exact test.

*** Bowker's test of symmetry.

During the pandemic, 28 (24.35%) patients changed their therapy, with the most common reasons for changing therapy being relapses—16 (61.54%) patients, side effects—5 (17.85%) patients (Table 1). There were no statistically significant differences in the reasons for changing therapy before and during the pandemic period. During the pandemic period, HET was used as subsequent therapy in 18 (64.23%) patients.

NEDA was found in 58 (50.43%) patients before the pandemic, and in 90 (78.26%) during the pandemic.

### 3.3 Infections Before and During the COVID‐19 Pandemic

There were no differences in the rate of non‐COVID infections before and during the pandemic—with infections found in 78 (67.82%) patients before the pandemic period, and in 64 (55.65%) during the pandemic, *p* = 0.16 (Table 2). Upper respiratory and throat infections were the most common both before and during the pandemic period. There were no differences in the course of infections in patients with MS compared to other members of their families (Table 2).

**Table 2:** Comparison of infection profiles and course in MS patients before and during the COVID‐19 pandemic.

Characteristic	Time point		*p*
	Up to II 2020, *n* = 115	III 2020 and later, *n* = 115	
Infections (other than COVID‐19)	78 (67.82%)	64 (55.65%)	0.16*
Upper respiratory and throat	46 (40%)	38 (33.04%)	0.55*
Herpes infections	8 (6.96%)	4 (3.48%)	0.49**
Pneumonia	7 (6.1%)	5 (4.35%)	0.84**
Gastrointestinal issues	6 (5.22%)	4 (3.48%)	0.81**
Urinary tract infections	23 (20%)	20 (17.39%)	0.88**
Other infections	1^1^ (0.09%)	2^2^ (1.74%)	0.84**
Serious infections	2 (1.74%)	1 (0.09%)	0.84**
Frequency of infectious diseases (compared to other family members)			0.060***
Normal	65 (56.52%)	74 (64.35%)	
More often than normal	31 (26.96%)	24 (20.87%)	
Less often than normal	18 (15.65%)	17 (14.78%)	

* *χ* ^2^ test.

** Fisher's exact test.

*** Bowker's test of symmetry.

^1^ Appendicitis.

^2^ Candidiasis (1 patient), eye infection (1 patient).

After vaccination, 15 (17.65%) patients experienced at least one relapse, MRI activity was observed in 13 (15.29%), and EDSS progression in 4 (4.71%). NEDA among vaccinated patients was observed in 69 (81.17%), while it was noted in 21 (70%) unvaccinated patients, *p* = 0.44.

### 3.4 Impact of COVID‐19 Vaccination

The characteristics of COVID‐19 vaccinations in the studied group are provided in Table 3. Before vaccination, 68 (59.57%) of MS patients experienced COVID‐19, compared to 56 (48.84%) after vaccination (Table 4). No severe cases were reported after vaccination, compared to two severe cases before vaccination. The proportion of mild cases increased from 50% to 66.67%, while moderate cases decreased from 39.29% to 23.81%. The severity of COVID‐19 courses showed a statistically significant improvement after vaccination, with more patients experiencing no symptoms or a mild course of COVID‐19—28/55 (50.9%) versus 31/43 (72.1%), *p* = 0.03. When compared to other family members, the relative severity of the COVID‐19 course in vaccinated MS patients remained stable (Table 4).

**Table 3:** COVID‐19 vaccination characteristics.

Characteristic	*N*	*n* (%)
Vaccination against COVID‐19	115	88 (76.52%)
Number of doses:	88	
One		3 (3.41%)
Two		31 (35.23%)
Three		45 (51.14%)
Four		9 (10.23%)
Year of the first vaccination:	88	
2020		14 (15.91%)
2021		32 (36.36%)
2022		38 (43.18%)
2023		4 (4.54%)

**Table 4:** Comparison of COVID‐19 incidence and severity in MS patients before and after COVID‐19 vaccination.

**Characteristic**	**Time point**		*p*
	**Before vaccination, *n*** = **88**	**After vaccination, *n*** = **88**	
COVID‐19	55 (62.5%)	43 (48.86%)	0.3*
Severity of the COVID‐19 course			
Symptomless	2 (3.63%)	4 (9.3%)	0.03**
Mild	26 (47.27%)	27 (62.79%)	
Moderate	21 (38.18%)	12 (27.91%)	
Severe	6 (10.9%)	0 (0%)	
Severity of the COVID‐19 course compared to other family members			
The same severity	29 (52.73%)	30 (69.77%)	0.41**
Less severe	15 (27.27%)	7 (16.28%)	
More severe	11 (20%)	6 (13.95%)	

* Mcnemar test.

** Bowker's test of symmetry.

### 3.5 Disease Activity Predictors

Among the predictors, the only statistically significant factor was disease activity before the pandemic period, which was associated with a significantly higher risk of relapse (odds ratio [OR] = 6.80, 95% confidence interval [CI]: 1.65–33.19, *p* = 0.010). Other predictors, including age, sex, disease duration, unfavorable prognostic profile at diagnosis, current medication, COVID‐19 infection, and other infections, did not significantly affect relapse occurrence, EDSS progression, or MRI activity in MS patients during the (post)pandemic period (Table 5).

**Table 5:** Results of the logistic regression model for relapse occurrence, EDSS progression, and MRI activity occurrence outcomes.

Predictors	Clinical relapse			EDSS progression			MRI activity		
	OR	95% CI	*p*	OR	95% CI	*p*	OR	95% CI	*p*
(Intercept)	0.03	0.00–0.15	< 0.001	0.04	0.01–0.15	< 0.001	0.05	0.01–0.24	0.001
Age (centered by median = 45.0 years)	1.01	0.95–1.08	0.698	1.05	0.98–1.14	0.206	0.98	0.92–1.04	0.465
Sex (male with female as ref.)	1.15	0.22–4.90	0.852	1.96	0.36–9.66	0.406	0.49	0.07–2.24	0.404
Infection during pandemic (yes or no as ref)	1.15	0.94–1.44	0.179	1.09	0.90–1.33	0.383	0.98	0.68–1.24	0.882
Disease duration (centered by Median = 4.0 years)	0.97	0.80–1.14	0.753	1.03	0.86–1.19	0.730	1.01	0.85–1.18	0.866
Current medication since pandemic (HETA as ref.)	0.39	0.02–2.95	0.429	0.86	0.04–7.07	0.901	0.74	0.10–3.71	0.734
Unfavorable prognostic profile at diagnosis (yes with no as ref.)	1.32	0.27–6.48	0.727	1.56	0.78–2.34	0.654	3.94	0.75–30.47	0.130
Disease activity before the pandemic (yes with no as ref.)	6.80	1.65–33.19	0.010	3.03	0.52–17.28	0.197	1.30	0.25–5.51	0.733
COVID (yes with no as ref.)	1.43	0.36–6.37	0.614	0.66	0.13–3.31	0.608	0.74	0.19–2.95	0.665
*R* ^2^_Tjur_	0.116			0.116			0.055		

*Note:* 95% CI, confidence interval 95%; OR, odds ratio; *p, p* value of statistical test.

## 4 Discussion

The main findings of this long‐term follow‐up of a cohort of RRMS patients treated with DMTs in the pre‐ and (post)pandemic periods show that the COVID‐19 pandemic did not negatively affect the course of the disease. The primary reason for changing therapy was relapse activity. In addition, we did not observe an increased risk of non‐COVID infections during the pandemic compared to several years prior, or an adverse effect of the COVID‐19 vaccination on the course of MS. In multivariate analysis, the independent factor associated with disease activity (relapses) during the pandemic was disease activity before the pandemic.

### 4.1 Treatment Change

Changing or discontinuing DMTs in MS patients is one of the challenges in clinical practice [21]. In the pre‐pandemic period, 56.52% of patients changed therapy, achieving disease inactivity in subsequent (pandemic) years of follow‐up. As a natural consequence of achieving disease stabilization in the study group during the first years of treatment (before the pandemic), there was a lower percentage of patients changing DMT in subsequent (pandemic) years and a higher rate of patients with NEDA.

During the COVID‐19 pandemic, a new and special situation has arisen, creating the potential for the emergence of an additional determinant affecting the need to change DMTs. To our knowledge, our publication is the first to evaluate such a long period of patient follow‐up during the pre‐ and (post)pandemic periods. Our results confirming a lack of impact of the pandemic on the course of MS are consistent with previous publications [18, 19]. Babtain et al. evaluated a 5‐year period of DMT treatment of MS patients, and found no adverse effect of the pandemic on the course of MS [18]. Salter et al., in a large longitudinal study evaluating the association of the COVID‐19 infection with the course of MS found that COVID‐19 infection was not associated with changes in symptom severity or disability [22].

In a study published by Apiwattanakul et al., there was no link between COVID‐19 or COVID‐19 vaccination and a higher frequency of relapse in patients with demyelinating diseases—including MS and neuromyelitis optica spectrum disorders [23]. Other publications have postulated the possibility of disease exacerbation, especially in patients with high disease activity not treated with HET. However, most of the available literature does not support the association of pandemic with the risk of disease exacerbation and progression [18, 19, 24, 25]. Initiation, sequencing, and change of therapy should not depend on the epidemiological situation. Indeed, the only DMTs requiring increased attention and patient monitoring are anti‐CD20 therapies [19, 20].

The primary reasons for changing DMTs in MS patients are disease activity and side effects [26, 27]. In the observed group of patients, this was the occurrence of relapses during therapy. The development of available DMTs and their increasing efficacy in inhibiting disease activity and progression is facilitating disease control and providing the opportunity to achieve NEDA in an increasing number of patients. Numerous studies suggest that HETs are more effective when used early in the course of the disease [28, 29, 30]. The availability of new HET therapies in recent years has increased the proportion of patients treated with HET, which we also observed in our cohort, despite concerns about the risk of infection and exacerbation of MS during the pandemic. Furthermore, we used all of the available HETs, despite concerns about modulation of the immune system and lower seroconversion efficacy to vaccination in patients treated with anti‐CD20 [31].

### 4.2 Infections

The current work compared the course of COVID‐19 and other infections between RRMS patients and their family members. We found no increased incidence of infection in RRMS patients and no differences in the course of COVID‐19 in RRMS patients compared to other family members. However, we must emphasize that the use of subjective data collection from patient interviews and the lack of an objective method for measuring patients' perception of infections in our study. This limitation may complicate the interpretation of the findings related to infection rates. A study by Levitz et al. found a benign course of COVID‐19 in more than 80% of patients with MS who developed COVID‐19. While this 1.7‐year follow‐up linked COVID‐19 infection to the possible risk of MS exacerbations, the association of COVID‐19 with the risk of disability progression was not confirmed, similar to our observation [17]. These observations support the thesis that the COVID‐19 pandemic had no significant effect on the increased risk of a worse course of MS or the risk of other infections in patients with RRMS.

### 4.3 COVID‐19 Vaccination

Our study confirmed the positive role of COVID vaccination in reducing the risk of disease and alleviating the course of COVID‐19 infection. Available data suggest that COVID‐19 vaccination not only reduces the risk of severe outcomes in MS patients but also shifts the disease course toward milder presentations, which is consistent with observations in our study [19]. These results should encourage clinicians to continue strong recommendations for COVID‐19 vaccination in MS patients, as the benefits of preventing severe COVID‐19 far outweigh any theoretical risks related to MS progression.

### 4.4 Disease Activity Predictors

The search for predictors of disease activity and progression, as well as biomarkers of response to applied DMT, is one of the more current research topics in the MS field [32, 33]. Widely recognized unfavorable prognostic predictors now make it possible to select appropriate therapy early in treatment [34, 35]. However, the unfavorable prognostic profile we identified in 31.3% of patients at the time of DMT initiation did not prove to be a factor associated with disease activity at the time of pandemic in the multivariate analysis. Nonetheless, disease activity at the time of initial DMT was found to be a predictor of disease activity in subsequent years, confirming the need for effective therapies from the very beginning of chronic immunomodulatory treatment.

### 4.5 Strengths and Limitations of the Study

The strengths of the study include the long duration time (8 years) of observation and real‐world clinical data collection. Our work included collecting data not only on disease activity, but also on the safety of therapy, including infections, and comparing the incidence of infections and the course of COVID‐19 with patient family members. Knowing that results of real‐world clinical follow‐up can facilitate therapeutic decision‐making in individual patients, our data comparing COVID‐19 incidence and severity before and after vaccination in MS patients provides insights into the effectiveness of vaccination in reducing disease burden and mitigating severe outcomes in this vulnerable population.

The study also had several limitations. First and foremost, the work involved the observation of patients treated at a single center, which limited the size of the cohort evaluated and the performance of the comparisons presented. The study only included patients with RRMS; we did not analyze patients with progressive forms of MS, as there is already evidence that progressive forms are associated with an increased risk of infection [36, 37]. In addition, unmeasured confounding factors could influence the study results, such as selection bias or recall bias for infection data. Furthermore, no comparisons were made based on the DMT used, the coexistence of comorbidities as factors that could affect disease activity, or the incidence of infections in the study group. The lack of such analyses, as well as the small number of categories compared, was due to the size of the group and the potential for bias when comparing groups with small numbers. Also, we did not evaluate the long‐term efficacy of vaccination by measuring SARS‐CoV‐2 antibodies in the serum of MS patients, though this will be the subject of a future paper.

## 5 Conclusions

The COVID‐19 pandemic did not affect the course of MS in this single‐center long‐term follow‐up. The only factor associated with disease activity during the pandemic period appeared to be disease activity in the pre‐pandemic period. Knowing the determinants of the disease course, including infection, will help select and stratify therapy for individuals with RRMS. COVID‐19 vaccination is not only safe but also does not appear to unfavorably affect the natural progression of MS, making it an essential tool in the comprehensive care of MS patients.

## Author Contributions

Katarzyna Maciejowska, Zofia Tarnawska, Zofia Nowakowska, Mateusz Glodny contributed to the acquisition of data. Przemyslaw Puz, Marta Konieczna contributed to the analysis and interpretation of data. Przemyslaw Puz and Anetta Lasek‐Bal contributed to drafting and revising the manuscript.

## Consent

Informed consent was obtained from all subjects involved in the study.

## Conflicts of Interest

M.K., K.M., Z.T., Z.N., and M.G. declare no conflicts of interest. P.P. and A.L.B. have accepted speaker and consulting fees from Merck, Biogen, Sanofi, Roche, and Novartis.

## Transparency Statement

The lead author, Przemyslaw Puz, affirms that this manuscript is an honest, accurate, and transparent account of the study being reported; that no important aspects of the study have been omitted; and that any discrepancies from the study as planned (and, if relevant, registered) have been explained.

## Data Availability

For data protection reasons, the authors cannot distribute the underlying data. Interested researchers may contact the corresponding author.
